# Assessment of interspecies and intergeneric gene flow for the GM *Jatropha curcas* event X8#34 with high oleic acid content in seed

**DOI:** 10.1080/21645698.2025.2470484

**Published:** 2025-03-05

**Authors:** Kasthurirengan Sampath, Zhang Shilu, Hong Yan, Yogendra Kr. Tripathi, Srinivasan Ramachandran

**Affiliations:** a Temasek Lifesciences Laboratory, 1 Research Link, National University of Singapore, Singapore; b JOil (S) Pte Ltd, 1 Research Link, National University of Singapore, Singapore; c School of Biological Sciences, Nanyang Technological University, Singapore

**Keywords:** Interspecies, non-GM Jatropha, outcross, transgene flow, spatial isolation, pollen load

## Abstract

GM Jatropha X8#34 was placed for transgene flow assessment in the open field trial on Semakau Island, Singapore, between 2015 and 2017 to evaluate the potential gene flow to its non-GM counterparts and related species. The trial featured the GM Jatropha event X8#34, which is characterized by high oleic acid content, marker-free, and a homozygous transgene. The study focused on cross-pollination from the GM event to non-GM plants, analyzing factors such as distance, wind and insects mediated transfer, using event-specific multiplex PCR analysis of F1 seeds. Pollen dispersal by wind was also assessed to understand the extent of distance traveled and pollen load. Our results showed the maximum observed transgene flow was 4.5%, occurring in non-GM plants located 2 meters in third quarter of 2016, average for four quarters is 2.57%. However, as the distance increased, the transgene flow decreased significantly, at 4 meters distance observed 0.8% in fourth quarter and an average 0.25%. Transgene flow was not observed beyond 4 meters. These results are consistent with the exponential decrease in Jatropha pollen dispersed and captured by traps over distance, with no pollen detected beyond 6 meters through wind dispersal. Furthermore, no intrageneric transgene flow was detected from GM Jatropha to *Jatropha integerrima*, nor intergeneric transgene flow to related weedy species such as *Euphorbia hirta*, *Phyllanthus niruri*, or *Ricinus communis* (Castor bean), under open-field conditions (2015–2017). The findings suggest that Jatropha pollination is primarily facilitated by short-distance foraging insects, or overlapping branches between adjacent trees enhances cross-pollination rate due to denser floral display, and attracts more pollinators. An adequate separation distance (>8 meters) is sufficient to prevent unintended transgene flow from GM Jatropha to non-GM Jatropha in Singapore ecological conditions. Additionally, transgene flow between GM Jatropha and related horticultural shrub (*Jatropha integerrima*) or intergeneric relatives like *E. hirta*, *P. niruri*, and castor bean is unlikely under open field conditions.

## 1. Introduction

*Jatropha curcas*, a member of the Euphorbiaceae family, is one of the most promising perennial biofuel crops, widely distributed across tropical regions.^1^ Jatropha is a monoecious shrub producing unisexual flowers, but some studies reported that Jatropha bears hermaphrodite flowers occasionally.^2^ Cymose type of inflorescence with yellowish-green flowers is arranged in terminal cymes. Normally, Jatropha male flowers start opening from the first or second day of the inflorescence life (13–19 days), while female flowers open later, with 60% of them opening from the third to fifth day,^1, 3, 4^ and this flowering cycle continues in tropical climate conditions, while in arid and semi-arid region twice per year. The fruits are yellow to brown in color and seeds ellipsoidal in shape and contain up to 40% oil, with this content varying depending on the diverse eco-geographical environments in which the plant grows. The fuel properties of Jatropha biodiesel depend on its fatty acid composition. Plant oils typically contain five main fatty acids: palmitate (16:0) and stearate (18:0) (saturated), oleate (18:1) (monounsaturated), and linoleate (18:2) and linolenate (18:3) (polyunsaturated).^5^ Biodiesel rich in monounsaturated fatty acids, especially oleate, offers superior ignition quality, low NOx emissions, and stability.^6^ In contrast, high polyunsaturated fatty acid content, such as linoleate and linolenate, reduces stability and increases oxidation, affecting the cetane number (CN) and emission characteristics. Thus, biodiesel from high-oleate plant oils is expected to have better fuel properties. To overcome this issue, Qu et al. (2012) developed high oleic acid content GM Jatropha has been developed using a marker-free RNAi silencing strategy.^7^ This GM Jatropha variety, designated as event X8#34, has been subjected to open field trial in Singapore to assess transgene flow. Prior to the commercial release of this GM event, it is essential to evaluate potential ecological consequences, including the movement of pollen from GM Jatropha, to ensure environmental safety. Like other crop genes, novel transgenes can also spread through pollen and seed dispersal to populations of related crops, weeds, and distantly related species.^8^ Pollen-mediated gene flow raises concerns over transgene escape and its ecological risks.^9–12^ A transgene can transfer from a GM crop to wild relatives, persisting through hybridization and introgression.^13^ This may enhance weed competitiveness, seed production, and abundance, altering wild population dynamic.^14, 15^ Typically, plants disperse genes via pollen and seeds, but the contribution of each mode to overall gene flow can be asymmetrical, varying across different temporal and spatial scales.^16^ Gene flow occurs among all sexually compatible plant species through natural hybridization. Transgene flow through wind or insect-mediated cross-pollination is of particular concern because pollen can easily facilitate cross-pollination under natural conditions, potentially leading to significant ecological impacts in conventional fields.^17, 18^

Several GM field studies show higher transgene flow detected at close proximity, declining exponentially with distance.^19–21^ There are instances where the unintended spread of transgenes raised safety concerns. In fact, gene flow from certain GM crops is often cited as a major environmental issue.^12, 22, 23^ Gene flow is a key evolutionary process, with similar questions and methodologies applied across hybridizing crop species.^24^ Ellstrand et al. (1996) reported frequent spontaneous hybridization among vascular plants is common.^25^ While less common, intergeneric hybridization has been observed in families like Solanaceae, Brassicaceae, and Poaceae.^26–29^

Therefore, this study was conducted to provide useful information for the better understanding of pollen movement from the X8#34 GM Jatropha to non-GM Jatropha under open field conditions in Singapore. It is known that *J. curcas* can self- and cross-pollinate, but there are no data on the frequency of cross-pollination in open field conditions, particularly concerning distance. Therefore, our experiments are designed to evaluate the spatial and temporal (across four quarters of 2016) transgene transmission from the X8#34 GM Jatropha to non-GM Jatropha at various distances and directions. In addition, gene flow was evaluated intrageneric species like ornamental Jatropha (*Jatropha integerrima*), is also a monoecious shrub with cluster of bright red colored separate male and female flowers on the same inflorescence, intergeneric weeds (*Euphorbia hirta*, *Phyllanthus niruri*), also a monoecious herbs, self-pollinating as well as pollinated through cross-pollination, mainly insects, which attracted by presence of nectar, and distant relatives like *Ricinus communis* (Castor bean) also a self-pollinating monoecious shrub, and wind-pollination observed as male flowers produce large amount of powdery pollens, this study designed to fill this critical knowledge gap.

## 2. Materials and methods

In this study, the marker-free T2 transgenic line X8#34 with high oleic acid content was used as the pollen donor. This line was developed by Qu et al. (2012) through the RNAi-mediated silencing of the *JcFAD2*-1 gene, targeting an 862 bp coding region to enhance oleic acid content in a seed-specific manner using the soybean 7S gene promoter.^7^ The initial T0 plants generated through this transformation were self-pollinated, and the resulting T1 plants were grown in a greenhouse, where they underwent PCR analysis and seed fatty acid profiling. The high oleic acid-producing, homozygous marker-free X8#34 GM Jatropha plants were then selected and self-pollinated to produce T2 seeds.

### 2.1. Experiment site and design

The field trial site located at 1°12’34“N, 103°46’46“E, Semakau Island approximately 7 kmSouth from Singapore Mainland.

**Figure 1.**
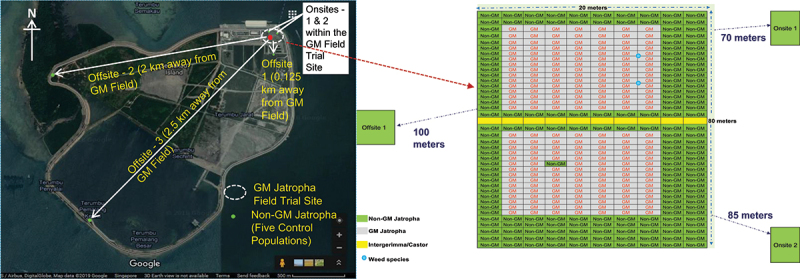
Aerial view of field with X8#34 GM Jatropha trial site at Semakau Island, Singapore. The GM field trial site represented in white dotted circle in the map and green dots with non-GM populations (offsite and onsite). Right side figure panel shows X8#34 GM field trial site in detail – gray colored blocks represent GM Jatropha and non-GM Jatropha planted at various range of distance (2, 4, 6, 8, and 10 meters), three non-GM *J. curcas* populations (2 onsite and 1 offsite) were highlighted in green colored blocks. *Jatropha integerrima*, castor and weeds species were planted in trial site, location as in the illustration below. This site is designed to evaluate the transmission of the transgene gene in small scale field trial.

The trial site was designed to assess cross-pollination from X8#34 GM Jatropha to non-GM Jatropha plants at various distances, as well as to evaluate potential transgene flow to interspecies (*J. integerrima*), other related species (weeds), and castor bean. Two adjacent X8#34 GM Jatropha plants (420 m^2^ each block; 2 meters spacing between plants); non-GM plants were planted at range of distance (2, 4, 6, 8, and 10 meters) and directions related to the X8#34 GM Jatropha plants. A non-GM plant was positioned in the center of the X8#34 GM Jatropha block to evaluate transgene flow at extreme transgenic pollen exposure. In addition, we placed five non-GM Jatropha populations that were planted at various distances away from the GM population to assess the possibility of gene flow via wind and insects over medium to long foraging distances. Two of these non-GM Jatropha populations (15 plants each) were established on the trial site, located 70 meters and 85 meters from the X8#34 GM Jatropha trial site and >100 meters apart from each other. Three additional non-GM Jatropha populations were planted at distances of 0.125, 2.0, and 2.5 km away from the GM trial site on Semakau Island (Figure 1).

A single row of vegetative clones of fertile *J. integerrima* and seed-derived castor (10 plants each) was planted 2 meters from the X8#34 GM Jatropha plants, along with common Euphorbiaceae weed species (*E. hirta*, and *P.niruri*) placed between GM Jatropha plants with 1 meter spacing, to evaluate potential interspecies and intergeneric transgene flow. (Figure 1).

### 2.2. Sampling procedure

#### 2.2.1. Testing outcrossing between event X8#34 GM Jatropha and non-GM Jatropha

Fresh seeds were harvested from selected non-GM Jatropha plants located within the GM Jatropha trial site, at distances ranging from 2 to 10 meters in various directions, as well as from control site populations. These control sites included two on-site populations (located 70 and 85 meters within the GM trial site) and three off-site populations (0.125, 2.0, and 2.5 km away from the GM trial site). The seeds were collected over four growth seasons, from Q1 2016 to Q4 2016, during which uniform flowering periods were observed between the GM and non-GM Jatropha populations. The harvested F1 seeds were germinated in a greenhouse, and germination efficiencies were recorded after the emergence of true leaves. Young leaves from individual non-GM Jatropha seedlings were harvested, immediately frozen in liquid nitrogen, ground into fine powder, and stored at −80°C in an eppendorf with proper labeling.

For the five control populations, five leaf samples from five seedlings were harvested, pooled together, and considered as one sample. As a positive control, a mixture of non-GM leaf samples with GM Jatropha leaf (in a 4:1 ratio) was prepared, with leaf sizes ranging between 8 and 10 mm, then frozen and ground into fine powder.

#### 2.2.2. Interspecies hybridization between event X8#34 GM Jatropha with *J.* *integerrima*

Fresh F1 seeds were harvested from naturally and artificial pollinated plants between Q3 2015 of Q4 2016. The seeds were germinated in the green house and germination efficiencies were recorded. Leaf discs, 8–10 mm in diameter, were collected from five plants and pooled together as one sample, resulting a total of 20 pooled samples. The samples were grounded using liquid nitrogen and stored at −80°C. As a positive control, leaf discs from four *J.* *integerrima* plants were mixed with one disc from GM Jatropha. DNA was extracted using extraction kit (Promega, USA) and its quality and concentration were detected using NanoDrop (ND 2000 Spectrophotometer, Thermo Fisher Scientific, USA).

#### 2.2.3. Intergeneric hybridization between event X8#34 GM Jatropha with their relatives

##### 2.2.3.1. Transgene flow with their weedy and distant relatives

In Singapore, two tropical weeds from Euphorbiaceae family, *E. hirta* and *P. niruri* are commonly observed. Possible intergeneric cross with GM Jatropha was evaluated under two scenarios: in the first experiments, two weeds (*E. hirta and P. niruri*) were put in close proximity to GM Jatropha under natural open field conditions and the second scenario with weeds (*E. hirta and P. niruri*) flowers artificial dusted with GM Jatropha pollens. Seeds from both weeds were periodically harvested from naturally and artificial dusted plants between Q3 2015 to Q4 2016 and sown on pots with potting mix to facilitate germination in green house at ambient temperature conditions. Leaf discs sized 8–10 mm were collected from five plants and pooled together in a single tube, resulting in a total of 20 pooled samples. These samples were frozen in liquid nitrogen and stored at −80°C. For the positive control, four leaf discs from each weed species were mixed with one leaf disc from GM Jatropha.

##### 2.2.3.2. Transgene flow to *Ricinus communis*

We experimentally evaluated the possibility of gene flow from the X8#34 Jatropha to castor bean in both open field condition and artificial dusting with X8#34 Jatropha pollens. Seeds were periodically harvested from naturally and artificial dusted plants between Q2 2016 and Q2 2017 and subsequently germinated in the green house; 100 seedlings were grown and maintained. Leaf discs sized 8–10 mm were collected from five plants and pooled together in a single tube, resulting in a total of 20 pooled samples. These samples were frozen in liquid nitrogen and stored at −80°C. For the positive control, four leaf discs from castor were mixed with one leaf disc from GM Jatropha.

### 2.3. PCR analysis to detect outcrossing

The extracted DNA samples were subjected to event specific multiplex PCR screening. The RBF/JCR primer pair was used to detect transgene insertion in non-GM Jatropha and in other relative species, while the SSR marker (151F/151 R) served as an internal control to amplify the genomes of *Jatropha curcas* and *Jatropha integerrima*. For other related species, species-specific primer pairs were designed to amplify the chloroplast genome as an internal control (Table 1). Each PCR reaction mix had a total reaction volume of 20 µl, consisting of 1X PCR buffer [50 mm KCl, 10 mm Tris HCl (pH 9.0), 0.1% Triton-X, 1.5 mm MgCl_2_], 0.25 mm dNTP (Promega, USA), 3.5 pmol of each primer (Integrated DNA Technologies (IDT, Singapore), 1 U of Taq polymerase and 50–100 ng of DNA samples.

**Table 1.** Primers used for transgene flow screening and genome of non-GM Jatropha, *Jatropha integerrima*, *Euphorbia hirta*, *Phyllanthus nirui* and *Ricinus communis.*

S. No.	Primer Pairs	Sequences (5’ − 3’)	Size (bp)	Targets
1	RBF	GGCATTTAGA CCTACATGGACGC	556	Detect X8#34 GM transgene insertion.^7^
	JCR	GGTTTACTTATCAAATGCGTTGCTTTG		
2	151F	CCAAACAGATGACAGCTGATAGCA	315	Detect Jatropha genus genome.^30^
	151 R	TAGGGAGCCCAATAAACCTCTCAC		
3	EHf1	ATGATATCGGAGGGCTTCGC	216	Detect chloroplast genome of *E. hirta* (Genebank accession # HQ645733.1).
	EHr1	GCGAAGGGTTTGGACCAATTT		
4	PNf1	CGGGTCCTTCTTGAGAGAAT	890	Detect chloroplast genome of *P. niruri* Genebank accession # GU441807.1).
	PNr1	CGTATGCTGCACGAGCATTT		
5	C1f	TGGCTTCAAAAGATGGGCCT	878	Detect chloroplast genome of *R. communis* (Genebank accession # LK021494.1).
	C1r	TTGCACACGGCTTTCCCTAT		

For screening outcrosses in non-GM Jatropha, the PCR cycle was an initial denaturation at 95°C for 3 min, followed by 5 cycles of denaturation at 95°C for 30 s, annealing at 63°C for 30 s, and extension at 72°C for 50 s. This was followed by 25 cycles of denaturation at 94°C for 30 s, annealing at 53°C for 30 s, and extension at 65°C for 50 s, and a final extension at 65°C for 5 min in MJ research thermal cycler (PTC 100). For other samples, PCR cycles were as follows: 95°C − 5 min, 35 cycles of, 94°C − 30 s, 55°C − 30 s, 72°C − 30 s, followed by final extension of 72°C 5 min in MJ research thermal cycler (PTC 100). PCR products were analyzed using 1% agarose gel electrophoresis stained with SYBR safe DNA gel stain (Thermo Fisher Scientific, USA).

### 2.4. Pollen traps

**Figure 2.**
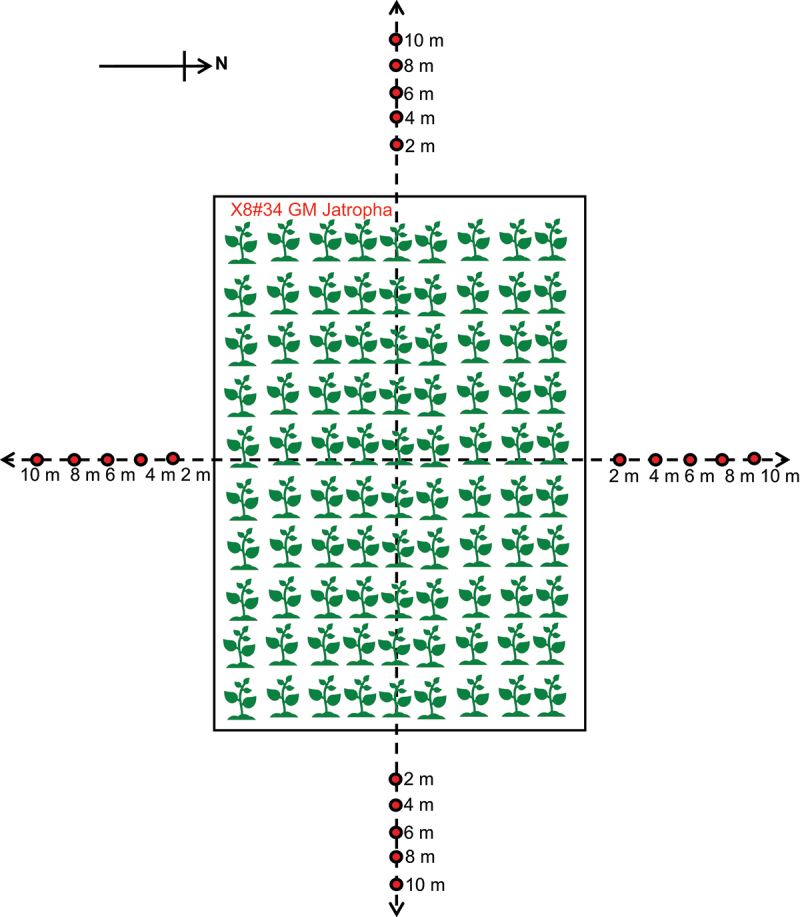
Illustration of pollen traps position in four directions (North, South, West, and East) and different distances from X8#34 GM Jatropha trial plot to capture pollen dispersed by wind, red dots indicate the position of pollen traps.

Pollen traps were strategically placed at varying distances (2, 4, 6, 8, and 10 meters) from the GM trial plot (Figure 2) in four directions (North, South, West, and East) to collect Jatropha pollen dispersed through wind during flowering period across four growth seasons in 2016. The physical positioning of the traps, along the wind speed and direction, was carefully considered when setting up the traps. Each pollen trap consisted of a pole with glass plate coated with Tween 20, mounted on a frame, with the height adjusted to match that of the flowering plants. The glass plate was set up in the morning and removed in the afternoon for five consecutive peak blooming days in each growth season, and inflorescences were tagged for the harvesting seeds. Pollen was retrieved from the glass plates by rinsing them with 1 M CTAB buffer (20 g/l CTAB, 1.4 M NaCl, 0.1 M Tris/HCl and 20 mm EDTA, pH adjusted to 8) and stored in 4°C.^31^ Number of Jatropha pollen was then counted using a hemocytometer under a light microscope. This experiment will provide us quantitative data for pollen transmitted via wind.

### 2.5. Insects/pollinators survey

Insect visitors survey was conducted between 2015 and 2016 at X8#34 GM Jatropha plants and non-GM plants at GM field trial site by scan sampling method.^32, 33^ Totally 26 species from 8 orders of insects were recorded in X8#34 GM trial site and non-GM Jatropha plant populations and the list were prepared with scientific and common names (Table S1).

Frequent insect visitors were recorded by walking through plant by plant for every 30 min from 0800–1630 hr in randomly at GM trial site (X8#34 GM and non-GM Jatropha plants) and non-GM populations for 20 days of blooming period, and repeated three times in 2016, to identify the potential insects that plays a role in cross-pollination. Insects were identified with the references available at Barcode of Life Datasystems (www.boldsystems.org).

### 2.6. Seed dormancy and viability under the natural conditions

Seeds of GM and non-GM Jatropha were collected during 2016–2017 in a single bulk harvest from fully matured fruits and mixed thoroughly. Three replicates of 100 seeds were randomly selected and placed on germination tray filled with potting mix and imbibed with 10 ml of sterile distilled water and incubated for three–four weeks at 25°C. Seed germination percentage were calculated as seed emerged with radicle and number of seeds sown, expressed in percentage. The germinated seeds were removed once they counted to avoid double counting. At the end of germination test, all the non-germinated seeds were collected and seed coats were removed to check the conditions of endosperms (dormancy).

In another experiments, ten plants were selected, five plants from each population (GM and non-GM Jatropha) to assess their seed viability under the natural conditions at Semakau Island, Singapore. Fruits were left out in the trees itself to assess their seed viability and germination (volunteering) capability. Assessment was carried out for three replicates in two seasons (rainy and dry).

## 3. Results and discussion

### 3.1. Testing outcrossing between event X8#34 GMJatropha and non-GM Jatropha plants

Mature fresh seeds were harvested from non-GM plants a various spatial distances covering four quarters in 2016. In total, more than 3,900 seeds were harvested from non-GM plants. The seeds harvested from non-GM population exhibited an average germination efficiency of 75%, with 2,900 seeds germinated. The resulting seedlings were analyzed for transgene presence to assess the extent of outcrossing from X8#34 GM Jatropha plants to non-GM Jatropha plants within GM trial site and at nearby onsite and offsite populations.

Overall, 2,750 non-GM samples were assessed for transgene transfer from GM Jatropha. Our event-specific multiplex PCR analysis confirmed that seeds harvested from non-GM plants located close to the GM Jatropha plants tested positive for the transgene, indicating localized outcrossing. The outcrossing rate varied between 0.8% and 4.5% across the four quarters, occurring within short distances of 2 and 4 meters.

**Figure 3.**
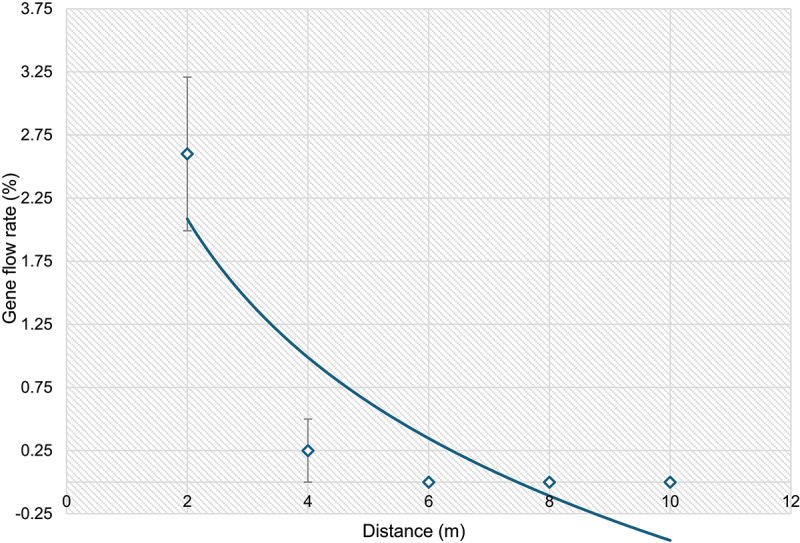
Rate of gene flow from X8#34 GM Jatropha to non-GM Jatropha at various distances from the GM Jatropha plants. Each data point represents the gene flow rate calculated from the average data for four quarters in 2016 (*R*^*2*^ = 0.78; *y* = − 1.582*x* +3.1812). The vertical error bars on data points represent the standard error of the mean.

In the first quarter, the average cross-pollination rate is 2.14% (6 out of 280 samples accessed), in second quarter average outcrossing is 2%, third and fourth quarter observe with 4.5% and 2.5% respectively at 2 meters spatial distance (Table 2; Figure S1). The highest outcrossing rate (10%) was observed in first quarter of 2016 from F1 seeds harvested from a non-GM surrounded on all four sides by GM Jatropha. In the subsequent three quarters, this plant exhibited outcrossing rates of 6%, 2% and 2% respectively (Figure S2 a-c). These results represent scenario where a non-GM Jatropha plant was exposed to the highest GM pollen intensity. The overall rate outcrossing rate from GM to non-GM plant at 2 meters isolation distance is 2.57% (18 out of 700 samples accessed) across four quarters in 2016. No transgene was detected in non-GM plants located at 4 meters away from GM plants for the first three quarters, while a low rate of transgene presence (0.8%) was observed in the fourth quarter (Table 2). No cross-pollination was detected beyond 4 meters from the GM Jatropha to non-GM Jatropha. A total of 19 non-GM were identified as hybrids containing the transgene out of 1,600 samples collected for populations at various distance within the GM trial site (outcrossing rate: 1.18%) (Table 2). The rates of gene flow from GM to non-GM obtained across four quarters of 2016 field trials were averaged, overall gene flow rates decreased when the distance from the GM Jatropha plants increased (*R*^*2*^ = 0.78) (Figure 3). No transgene flow was detected in non-GM plants located in five control sites, which included two onsite non-GM populations (70 m and 85 meters from GM field trial) (Table S2; Figure S3 a-b), as well as three offsite populations (offsite 1 at 0.125 km, offsite 2 at 2.0 km, and offsite 3 at 2.5 km from the GM trial site) (Table S2; Figure S4 a-c). Under current field trial ecological conditions, no cross-pollination was observed beyond 4 meters GM Jatropha and non-GM Jatropha.

**Table 2.** Rates of outcross from X8#34 GM Jatropha to non-GM Jatropha plants at a range of distance in four quarters in 2016.

Distance from GM plants (m)	2016				Average outcross rate
	Q1	Q2	Q3	Q4	
2	6/280 (2.14%)	3/150 (2.0%)	5/110 (4.5%)	4/160 (2.5%)	18/700 (2.57%)
4	0/75	0/75	0/125	1/125 (0.8%)	1/400 (0.25%)
6	0/50	0/50	0/50	0/50	0/200
8	0/25	0/25	0/25	0/25	0/100
10	0/25	0/25	0/75	0/75	0/200
Total	6/455 (1.32%)	3/325 (0.92%)	5/385 (1.30%)	4/435 (0.92%)	19/1600 (1.18%)

Our findings on the decline in transgene flow over distance are consistent with those observed in other GM crops. McPartlan and Dale (1994) reported that transgene flow in potatoes occurred primarily at short distances.^34^ When transgenic and non-transgenic potato plants were planted in alternate rows (with leaves touching), 24% of seedlings from non-transgenic plants were kanamycin resistant. At a distance of up to 3 meters, the resistance frequency was 2%, at 10 meters, it was 0.017%, and at 20 meters, no resistant progeny was observed. Similar trends were reported in oilseed rape, where cross-pollination was detected over very short distances, with successful pollination declining exponentially with increasing distance from the pollen source. Pollen from oilseed rape only occasionally traveled several hundred meters.^19^ Another study on transgenic *Brassica napus* concluded that increasing spatial isolation was more effective in reducing GM outcrossing than using a pollen barrier.^20^ In maize, studies have shown cross-pollination over distances, with Ma et al. (2004) detecting up to 82% cross-pollination at extremely close proximity, less than 1% at 28 meters, and recommending a 30-meter spatial isolation to maintain an acceptable outcrossing level (≤1%).^21^ Viljoen and Chetty (2011) observed in GM maize case study in South Africa, that a 45-meter isolation distance minimized cross-pollination between 1% and 0.1%, while a theoretical 135-meters distance was necessary to ensure a cross-pollination level between 1% and 0.1%, 503 meters for a level between <0.1% and 0.01%, and 1.8 km for a level between <0.01% and 0.001%.^31^ Luna et al. (2001) reported that maize cross-pollination occurred at a maximum distance of 200 meters from the source and recommended a 300-meters spatial isolation.^35^ In cotton, pollen-mediated transgene flow rates were always low (<1% of seeds at the field edge), even in fields near *Bt* cotton fields.^36^ In potatoes, a 20-meters isolation distance was adequate to prevent transgene flow.^34, 37^ Self-pollinating crops like soybean, the detectable gene flow overall rate was 0.02% in South Korea herbicide resistant GM soybean field trials conducted in 2014 and 2015, presence observed up to 3 meters distance in 2014 with 0.036% and 8 meters distance in 2015 with 0.034%,^38^ Yoshimura et al. (2006) reported that gene flow occurred up to 7 meters distance for glyphosate resistant soybean, this distance varied yearly (0% and 0.068%).^39^ But in Brazil, gene flow was found up to 10 meters for herbicide resistance soybean.^40^ Interestingly, the GM soybean event with recombinant proteins showed overall gene flow 0.32% for 3 varieties at 0.5 meter and observed up to 13 meters (0.085%) in one variety and other two varieties with 0% under South Korea conditions, confirms that variety also a factor in gene flow rate.^41^

From previous GM field trials, data show that crop-to-crop gene flow rate and spatial distance from transgenic and non-transgenic field may varies depending on ecological conditions. Transgenic crops distance standards or isolation distances are the minimum distance between GM and non-GM crops to be maintained, to minimize risk of gene flow and contamination. For instance, in Mexico, an isolation distance of 20 meters is recommended to maintain cross-fertilization below the threshold level for maize.^42^ In contrast, in the Europe Union, mandatory isolation distances for maize range from 15 to 800 meters in maize to prevent transgene contamination, for organic maize farms requires greater separation distances, typically 250 to 800 meters countries such as Denmark, Hungary, Luxembourg, the Netherlands, and Spain.^43–45^ In China, while isolation distances for GM maize and non‐GM maize are not officially regulated, a reference isolation distance of 300 meters has been proposed under agricultural GMO safety supervision policies.^46^ For self-pollination crops like soybean, a minimum isolation distances of 5 meters are required in the EU for different varieties; similar varieties 1 meter distance is sufficient.^47^

**Figure 4.**
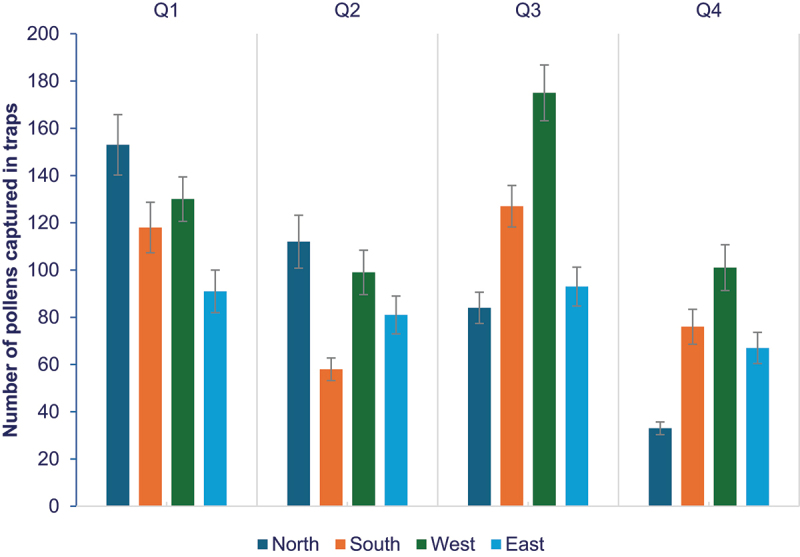
Summary of number of pollens captured in pollen traps at different directions (North, South, East, West) at various distances in four quarters of 2016. The vertical error bars on data points represent the standard error of the mean.

Our outcrossing data are consistent with pollen deposition data, which shows an exponential decrease in pollen deposition over distance. The highest number of pollens were captured at 2 meters, with numbers dropping as distance increased. Pollen deposition declined sharply between 4 and 6 meters, with no pollen captured beyond 6 meters. A total of 505 pollens were deposited in the western direction, coinciding with the highest cross-pollination evidence, while the lowest pollen load (332 pollens) was observed in the eastern direction (Figure 4). In the first two quarters of year 2016, wind dominated in northerly direction, the highest pollen load over the 5 days of pollen capture at the northern direction (265 pollen) followed by 229 number of pollens in western direction, this data party agrees with greatest incidence of wind direction. Wind direction in the third quarter is on southerly direction, the highest number of pollens captured in western direction (175 pollen) and lowest in northern direction with 84 pollens. In the fourth quarter, wind toggled between southerly and northerly direction, highest number of pollen load in western direction (101 pollen), and north with lowest number of pollen (33).

**Figure 5.**
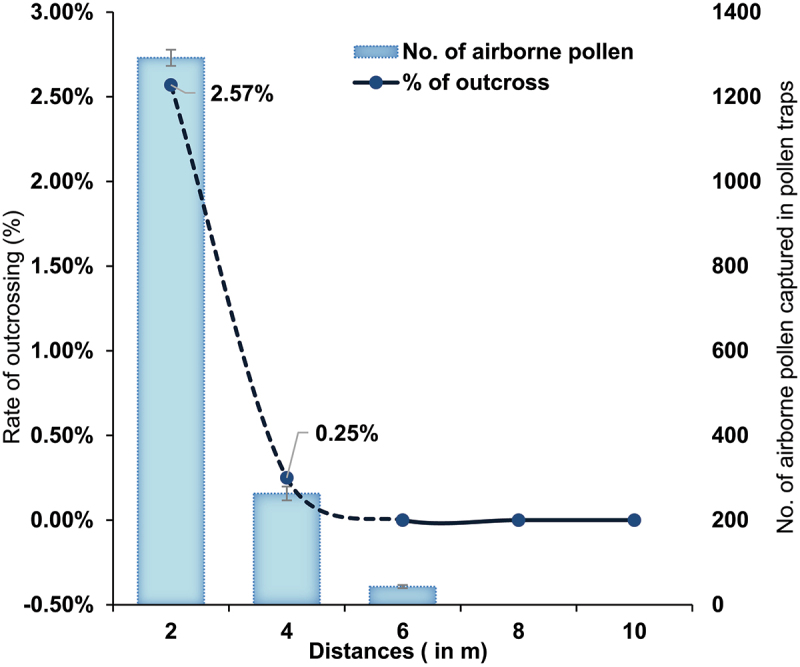
Comparison of pollen load and outcrossing rate for 5 days of flowering periods in all four quarters of the year 2016 at various spatial distances (2, 4, 6, 8, and 10 meters). Total number of airborne pollens captured in traps at various distances and transgene flow to non-GM plants over the same period. The vertical error bars on data points represent the standard error of the mean.

Pollen movement data collected during the four quarter in the year 2016 revealed that the highest pollen load was captured in the western direction (505 pollens), while the lowest was in the eastern direction (332 pollens). Pollen data for four quarters in year of 2016 for 5 days over flowering period showed highest extent of cross-pollination at a distance of 2 meters, where the overall average rate of outcrossing detected in plants was 2.57%. At this distance, 1,292 airborne pollens were captured in pollen traps. Cross-pollination declined sharply at 4 meters, where 0.25% of plants showed transgene presence, and 263 pollens were deposited in traps. At 6 meters, 43 pollens were captured, but no cross-pollination was detected (Figure 5). Beyond 6 meters, no airborne pollen was detected, likely pollen drops due to its dense and sticky nature. The highest percentage of cross-pollination was observed in non-GM plants spatially close to X8#34 GM Jatropha, i.e., up to 4 meters isolation distances. Thus, from the data, it is evident that there is relationship between airborne pollen load by wind dispersal and spatial isolation distance of non-GM on the extent of cross-pollination in this field trial.

Sears and Stanley-Horn (2000) studied the distance, direction, and density of *Bt* maize pollen dispersal at several field sites in Ontario, Canada, and found that most pollen fell within 5 meters of the field’s edge, regardless of direction.^48^ Similarly, Lavigne et al. (1998) suggested that approximately 50% of pollen produced by an individual plant falls within 3 meters radius, with outcrossing probability decreasing exponentially beyond this range in oilseed rape.^49^ Timmons et al. (1995) observed that airborne pollen levels declined with distance, with significant day-to-day fluctuations suggesting that oilseed rape pollen disperse rapidly from the source and does not remain airborne for extended periods.^50^ Studies on transgenic herbicide-resistant oilseed rape have consistently shown that pollen-mediated cross-pollination pre-dominantly occurs within a short distances, typically less than 10 meters from the source.^51^ Our experiments, incorporating wind data, pollen load measurements, isolation distances, and PCR analysis, suggest that airborne pollen load and wind direction are not only the factors influencing cross-pollination. Previous research reported that *Jatropha curcas* is self-compatible, with self-pollination fruit set rates ranging from 52.17% to 93.2%.^3, 52, 53^ The results from outcrossing shows that wind is not alone considered as a contributing factor for outcrossing.

**Figure 6.**
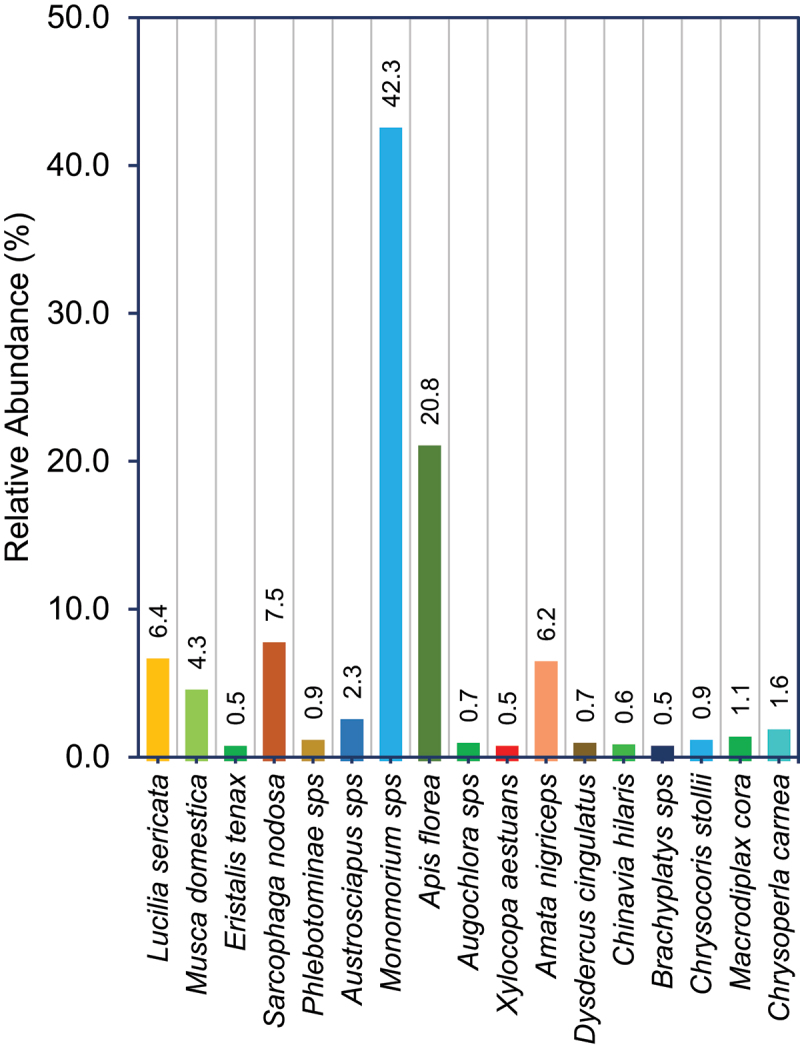
Percentage of insect diversity in X8#34 GM Jatropha trial site with GM and non-GM Jatropha populations were observed for 20 days of blooming period in four quarters of year 2016 in Semakau Island, Singapore.

**Figure 7.**
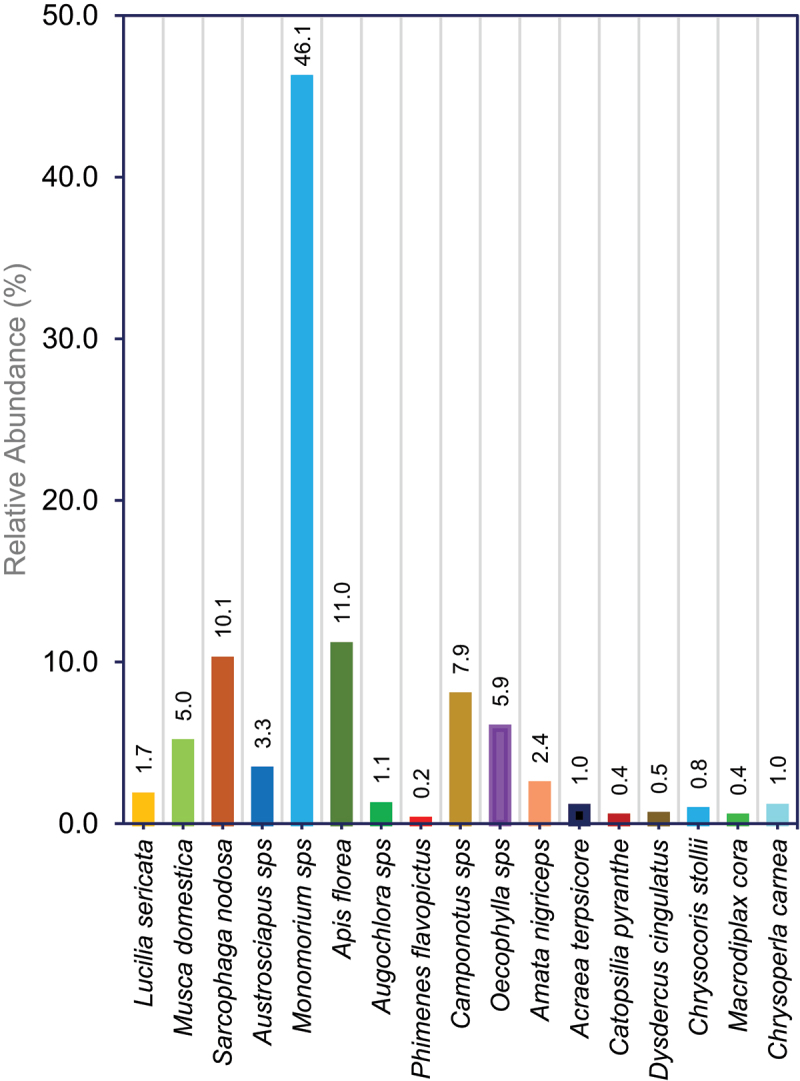
Percentage of insect diversity in control sites with non-GM Jatropha populations were observed for 20 days of blooming period in four quarters of year 2016 in Semakau Island, Singapore.

From our insect visitors survey, we have identified pollinators such as short distance foraging insects, is also a crucial factor for localized transgene flow in Jatropha. In general, *Jatropha curcas* is primarily pollinated by bees, wasps and ants serving as the main pollinators. From 26 insect species, 17 species are considered as frequent flower visitors for nectar and also serve as a pollinator in Jatropha, the rest of the 9 species just visited the plants but never contacted inflorescences during our observation windows. Highest number of insect species is observed in order Hymenoptera with 8 species followed by Diptera possess 6 insect species, Hemiptera with 4 species, Lepidoptera with 3 species, Odonata with 2 species and rest three orders (Neuroptera, Araneae, Coleoptera) with single insect species (Table S1). In our observation, *Monomorium* (ant) species from order Hymenoptera were predominant flower visitors in the GM Jatropha trial site (X8#34 GM and non-GM Jatropha populations) as well as in control populations, and always found throughout every observation, with highest relative abundance (42.3%) in the GM trial field and 46.1% in non-GM populations (Figures 6 and 7). *Apis florea* (Honeybee) are the next frequent flower visitors in GM trial site with abundance of 20.8% and 11.0% in non-GM populations (Figures 6 and 7). Our observation aligns with previous studies, which identified ants as the most frequent visitors to Jatropha flowers.^54–56^ We observed that insects played a significant role in transferring pollen between Jatropha plants, complementing wind-mediated pollination. Similarly, in soybean, Yoshimura (2001), confirmed that wind is not a sole factor for pollination, insect pollinators largely contribute toward cross-pollination, mostly by honeybees,^57^ *Apis mellifera*.^58–60^ During our field trial, we observed a lower abundance of medium- and long-range foraging insects, which likely contributed to the absence of transgene outcrossing in non-GM populations beyond 4 meters. Based on pollen dispersal patterns and insect visitors’ pollinators survey, maintaining adequate isolation distance is crucial factors to minimize/avoid the transgene flow from the GM to non-GM Jatropha, and also to consider the species’ reproductive biology. Our findings could be a baseline to establish the isolation distance for GM Jatropha, however the required isolation distance may vary depending on traits being placed for trials, ecological conditions, and specific environmental factors.

### 3.2. Seed dormancy and viability under the natural conditions

Seeds with 10–12% moisture content were used for germination tests, to the check dormancy. Both GM and non-GM Jatropha seeds were incubated 25°C for three–four weeks to assess their germination percentage and dormancy. Around 75–80% of seeds protruded with radicle in both populations from 5th day onwards (average 15%), and the peak seed germination period was observed between 7th and 15th days (average 28% and 75%), after 21st days there is no germination occurred, non-germinated seeds were collected, and seed coat removed, observed that endosperm were rotten/deteriorated. There is no significant difference in seed germination percentage between GM and non-GM seeds. In another scenario, under natural conditions, allowed matured fruits to be attached with the inflorescence stalk on tree itself until yellowish to brown in color unless absence of heavy rains. We have observed that fruits usually drop down during the rainy days. When they contact with the ground, fruit shells starts to disintegrate to facilitating seed germinations. Around 65–70% of the fruits exhibited with protrusion of radicle from seeds, germination started within a couple of weeks underneath the trees, and the rest were decayed or deteriorated. Fruits are not dispersed through wind/insects due to bigger in size and weight.

During dry season (temperature between 30 and 34°C, RH 75–85%), brown dried fruits were dropped down with intact seeds, within two to four-week fruits deteriorated due to environmental stress, insects and consequently affects the seed germination and vigor. Around 15% of fruits seeds possess complete endosperm and observed another 10% seeds with partially discolored endosperm; both the seeds were allowed to germinate on suitable conditions at 25°C with 80–85% RH. Seeds with intact endosperms germinated completely and discolored endosperms failed to germinate even though conditions are suitable for gemination. It shows that seeds exposed to the natural conditions undergo deterioration/viability loss faster than the seeds stored in controlled environment. For oil seeds like *Jatropha curcas*, the moisture level is detrimental as this favors fungal pathogen to deteriorate their physiological nature.^61^ Seeds will lose their viability if fruits are not harvested on time and inappropriate processing conditions. Windauer et al. (2011) studied the effects of temperature on dormancy, at 25°C incubation a fraction of seeds expressed absolute dormancy, and seeds incubated above 25°C increase the rate of induction of secondary dormancy, despite seeds incubated at 30°C showed rapid germination with less percentage.^62^ From our observations, we understood that Jatropha seeds very unlikely undergoes dormant stages, so there might be less chance for volunteer or feral populations post field trials.

### 3.3. Interspecies hybridization between *J.* *curcas* with *J.* *integerrima*

We examined the possibility under two scenarios: natural and artificial pollination. For the first scenario, *J. integerrima* plants were planted adjacent to GM Jatropha were allowed to flower at GM Jatropha flowering period and set seeds naturally. In the second scenario, around 500 newly opened *J. integerrima* flowers from tagged inflorescences were artificially dusted with GM Jatropha pollens. The seeds produced were morphologically similar. After germination in the greenhouse, naturally pollinated seeds had >50% and artificially pollinated seeds had around 38% germination rate. The germinated seedlings were morphologically similar with *J. integerrima* plants. Event-specific PCR analysis revealed absence of the transgene in any seedlings, indicating no interspecies hybridization between *J. integerrima* and *J. curcas* (Figure S5 a-b). We conclude that *J. curcas* cannot serve as a pollen donor to *J. integerrima* under open field conditions, which aligns with the findings of earlier study.^63^ They observed that while the pollen tube of *J. curcas* entered the ovule of *J. integerrima*, fertilization was unsuccessful.

Several studies have documented that successful crossing occurs only when *J*. *curcas* is the female parent.^63–66^ Inter-species cross-pollination among Jatropha species is generally challenging. For example, Kumar et al. (2009) reported signs of incompatibility in *J. curcas* x *Jatropha gossypifolia* cross-pollination, where the pollen tube reached the ovary but failed to produce seeds.^67^ Similarly, in *J. curcas* x *Jatropha podagrica* cross-pollination, bulged pollen tubes with reverse growth direction were observed, and in *J. curcas* x *Jatropha villosa*, crinkled and twisted pollen tubes failed to reach the ovary.^67^

Our findings confirm the absence of transgene flow from GM *J. curcas* to its close relative *J. integerrima*, which is commonly planted as a roadside in Singapore as an ornamental plant. Similar studies in other crop species to evaluate the possibility of transgene flow and the results were variable among different plants. For instance, interspecies and intraspecies transgene flow from GM canola to wild species has been documented by numerous researchers, raising concerns about the potential escape of transgenes and their introgression into the environment.^68–72^ Conversely, in herbicide-tolerant potato trials, no evidence of transgene interspecies hybridization was found in progenies of two Solanaceous weed species (*S. nigrum* and *S. dulcamara*) grown near GM plots.^34^ Even when researchers hand-dusted potato pollen onto these weeds to test for hybridization, no berries were formed in *S. dulcamara*, and no seeds were observed in berries of *S. nigrum*.^73^

### 3.4. Intergeneric hybridization between event X8#34 Jatropha with their relatives

#### 3.4.1. Testing out crossing with their wild relatives

Natural hybridization within the same plant species is a common occurrence in the plant kingdom, while sexual incompatibility with distant relative species due to genetic barriers is also well-documented. Intergeneric hybridization between Euphorbiaceae weeds that are not of the Jatropha genus and *Jatropha curcas* is theoretically improbable but has not been experimentally verified. Some of these weeds have a widespread distribution in the tropics, and their weediness raises concerns about the potential environmental impact of hybrids with *J. curcas*. The possibility of intergeneric cross-pollination with GM Jatropha was evaluated, and two weed species (*E. hirta* and *P. niruri*) were placed in close proximity to GM Jatropha under natural pollination and artificially dusted under open field conditions. The harvested seeds from both *E. hirta* and *P. niruri* exhibited normal morphology, with tiny seeds identical to those typically produced by these species. After germination, the seedlings were transplanted into individual pots, and their overall phenotypes were consistent with those of the mother plants. Event-specific multiplex PCR analysis provided no evidence of intergeneric hybridization between *J. curcas* and these two weedy species. In the PCR analysis of *E. hirta* and *P. niruri*, only the weed samples intentionally spiked with X8#34 GM Jatropha leaf sample showed amplification of the transgene. All other samples exclusively amplified the weed-specific genome fragments (Figures S6 a-b; Figure S7 a-b). This suggests that intergeneric hybridization between GM Jatropha and these weedy species did not occur under the tested conditions, this shows that there is no successful hybrid seeds produced.

#### 3.4.2. Testing out crossing with *Ricinus communis*

Castor bean (*Ricinus communis*), a commercial and community crop in the tropics, also belongs to the Euphorbiaceae family. Laosatit et al. (2017) reported that direct and reciprocal intergeneric crosses between *Ricinus communis* and *Jatropha curcas* are generally unsuccessful due to post-fertilization incompatibility. Reported that these crossability barriers could be overcome by *in* *vitro* culturing of fertilized ovules to produce hybrids.^74^ We experimentally evaluated possibility of gene flow from GM Jatropha to castor bean under natural and artificial dusted in an open field condition. Seeds were harvested from castor bean plants from both populations and germinated in the greenhouse. The resulting seedlings were phenotypically similar to their mother plants. Event-specific multiplex PCR analysis revealed no evidence of transgene hybridization between GM Jatropha and castor bean. In the PCR analysis, only samples intentionally spiked with GM Jatropha were positive for both the transgene and a castor chloroplast reference gene. All other samples were negative for the transgene but positive for the chloroplast reference gene (Figure S8 a-b). Field observations also noted a few aborted flowers, some fruits without endosperms, and harvested seeds that were morphologically similar to castor bean. This aligns with Laosatit et al. (2017), who also reported a lack of endosperm in direct and reciprocal crosses between Jatropha and castor.^74^ These findings indicate that the intergeneric hybridization barrier between castor and Jatropha is primarily post-fertilization. Previous study by Reddy et al. (1987) also reported strong cross-incompatibility between *J. curcas* and castor.^75^ Additionally, in reciprocal crosses, Jatropha pollen often failed to germinate, resulting in either coiled or spathulate tips. Another study on intergeneric crosses between castor and six Jatropha species found that most crosses failed due to incompatibility between pollen-pistil recognition mechanisms, which arrested further pollen development.^76^ In contrast, Gedil et al. (2009) successfully obtained intergeneric hybrid plants in both direct and reciprocal crosses between *Ricinus communis* and *Manihot esculenta* by using an embryo rescue technique.^77^ This highlights the complexity of intergeneric hybridization and the need for advanced techniques to overcome natural reproductive barriers.

## 4. Conclusion

This study comprehensively assessed transgene flow from X8#34 GM Jatropha with high oleic acid content to non-GM Jatropha across varying spatial distances across four quarters in 2016. By examining pollen dispersal patterns and insects pollinators abundance, we conclude that intraspecies transgene flow via cross-pollination from GM Jatropha to non-GM Jatropha is possible, but only under specific conditions. Transgene flow occurs when flowering period overlaps, plants are in close proximity (≤4 meters), and presence of short foraging insects under Singapore ecological conditions. Our findings demonstrate that appropriate crop spacing can mitigate unintended transgene flow to conventional Jatropha. In interspecies experiments, no transgene flow was observed from X8#34 GM Jatropha to ornamental species (integerrima), experimentally verifying their sexual incompatibility. Furthermore, there is no intergeneric gene flow to weedy species and a distant relative, castor bean in natural and artificial dusted pollination conditions in open field conditions. Overall, our findings demonstrate that X8#34 GM *Jatropha curcas* poses minimal risks of transgene dissemination through pollen- and seed-mediated, insects-facilitated transgene flow to non-GM counterparts, while presenting no risks of transgene transfer to other Jatropha species, and other relative weedy plants in open field environments.

## Supplementary Material

Supplemental materials_Kasthurirengan et al Jatropha_clean.docx
