# Supplementary material for: Assessment of interspecies and intergeneric gene flow for the GM Jatropha curcas event X8#34 with high oleic acid content in seed
Source: GM Crops Food. 2025 Mar 5;16(1):235–51. doi: 10.1080/21645698.2025.2470484 (PMC11901391; doi:10.1080/21645698.2025.2470484)
Supplement: Supplemental materials_Kasthurirengan et al Jatropha_clean.docx [file KGMC_A_2470484_SM5073.docx]

**Supplemental material for:** Kasthurirengan Sampath *et al.* Assessment of interspecies and intergeneric gene flow between the event X8#34 GM *Jatropha curcas* with oleic acid content in seed.

**Section 1: Table**

**Table S1:** List of insect species observed in X8#34 GM trial site and non-GM populations between 2015 and 2016 in Semakau Island, Singapore

| Order | Insect Names | | Presence/Absence | |  |
| --- | --- | --- | --- | --- | --- |
|  | Binomial | Common | X8#34 GM Jatropha trial site | non-GM Jatropha populations | |
| Diptera | *Lucilia sericata* | Green bottle fly | + | + | |
|  | *Musca domestica* | House fly | + | + | |
|  | *Eristalis tenax* | Drone fly | + | + | |
|  | *Sarcophaga nodosa* | Flesh fly | + | + | |
|  | *Phlebotominae sps* | Sand flly | + | + | |
|  | *Austrosciapus sps* | Green long-legged fly | + | + | |
| Hymenoptera | *Monomorium* sps | Ants | + | + | |
|  | *Apis florea* | Red dwarf honeybee | + | + | |
|  | *Augochlora* sps | Sweat bee | + | + | |
|  | *Xylocopa aestuans* | Carpenter bee | + | + | |
|  | *Scolia* sps | Flower wasp | + | - | |
|  | *Phimenes flavopictus* | Wasp | - | + | |
|  | *Camponotus sps* | Ants | - | + | |
|  | *Oecophylla sps* | Ants | - | + | |
| Lepidotera | *Amata nigriceps* | Moth | + | + | |
|  | *Acraea terpsicore* | Tawny coster | + | + | |
|  | *Catopsilia pyranthe* | Mottle emigrant | + | + | |
| Hemiptera | *Dysdercus cingulatus* | Red cotton stainer | + | + | |
|  | *Chinavia hilaris* | Green stink bug | + | + | |
|  | *Brachyplatys* sps | Shield bug | + | - | |
|  | *Chrysocoris stollii* | Jewel bug | + | + | |
| Odonata | *Macrodiplax cora* | Dragon fly | + | + | |
|  | *Pseudagrion microcephalum* | Damselfly | - | + | |
| Neuroptera | *Chrysoperla carnea* | Lace wing | + | + | |
| Araneae | *Oxyopes birmanicus* | Lynx spider | + | - | |
| Coleoptera | *Cheilomenes sexmaculata* | Lady bug | + | + | |

**Table S2:** Rates of outcross from X8#34 GM Jatropha to non-GM Jatropha (five populations) at a range of distance in four quarters in 2016.

| Distance from the GM trial site | 2016 | | | | Average outcross rate |
| --- | --- | --- | --- | --- | --- |
|  | **Q1** | **Q2** | **Q3** | **Q4** |  |
| 70 m | 0/75 | 0/50 | 0/75 | 0/50 | 0/250 |
| 85 m | 0/75 | 0/50 | 0/75 | 0/50 | 0/250 |
| 125 m | 0/75 | 0/50 | 0/75 | 0/50 | 0/250 |
| 2.0 km | 0/75 | 0/50 | 0/75 | 0/50 | 0/250 |
| 2.5 km | 0/75 | 0/50 | 0/75 | 0/50 | 0/250 |
| Total | 0/375 | 0/250 | 0/375 | 0/250 | 0/1250 |

**Section 2: Figures**

**Figure S1:** Rate of outcrossing detected at 2 meter and 4 meter in non-GM populations in four quarters of 2016. The vertical error bars on data points represents the standard error of the mean.

**Figure S2:** Multiplex transgene screening by event specific transgene primers (JCR/RBF) and an internal control primer pair (151F/R). a) PCR results of DNA samples from seedlings from the non-transgenic plant surrounded by X8#34 GM Jatropha plants in four sides, b) PCR results for seedlings from one non-transgenic plant 2 meter away from X8#34 GM Jatropha. * denotes out crossed DNA samples, 100 bp NEB ladder were used as the size marker.


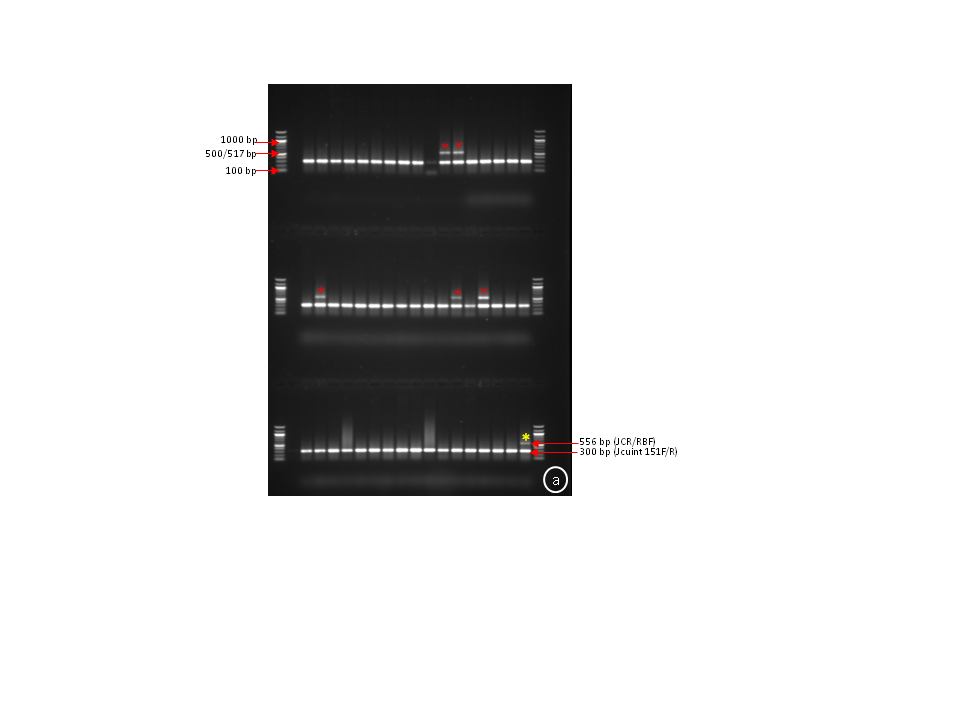

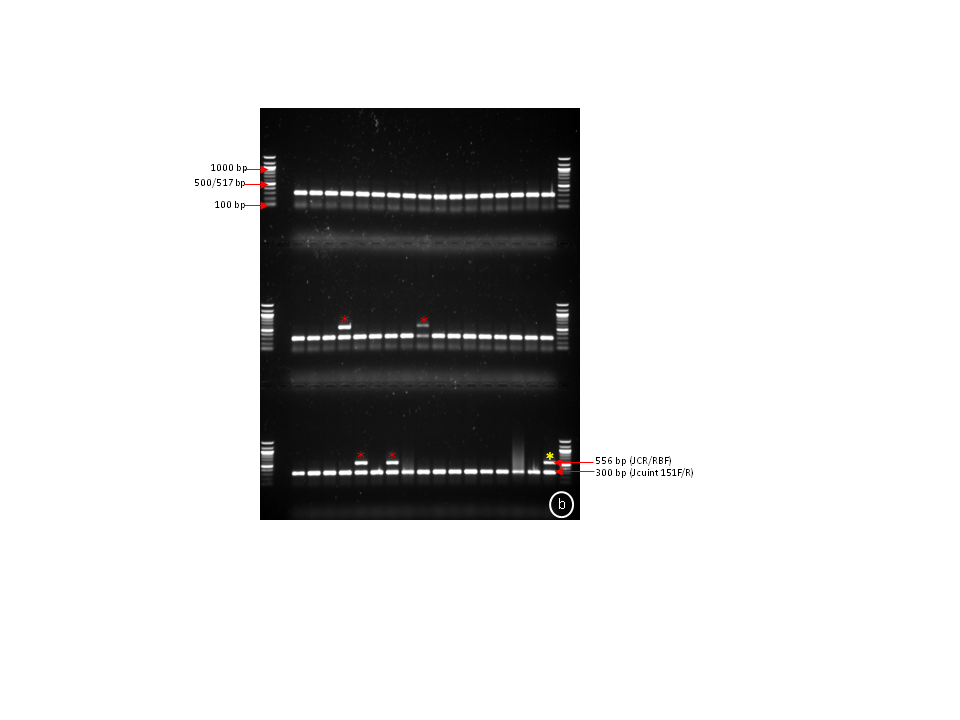


**Figure S2c:** Summary of outcross rate at 2 meter distance non-GM populations for four quarters in 2016. One non-GM surrounded by X8#34 GM plants in all four directions shows the extreme conditions with highest GM pollen load - highlighted in green, exhibits outcross in all quarters of 2016.

**Figure S3:** Multiplex transgene screening in seedlings from onsites non-transgenic plants by multiplex PCR with event specific primers, (JCR/RBF) and with internal control primers ( 151F/R) to amplify *J. curcas* genome. a) PCR results for seedlings derived from non-GM plants Onsite 1, 70 meter away from GM trial site, b) PCR results for seedlings derived from non-GM plants Onsite 2, 85 meter away. * denotes positive control sample (non-transgenic spiked with X8#34 GM Jatropha leaf samples), 100 bp NEB DNA ladder was used as the size marker.


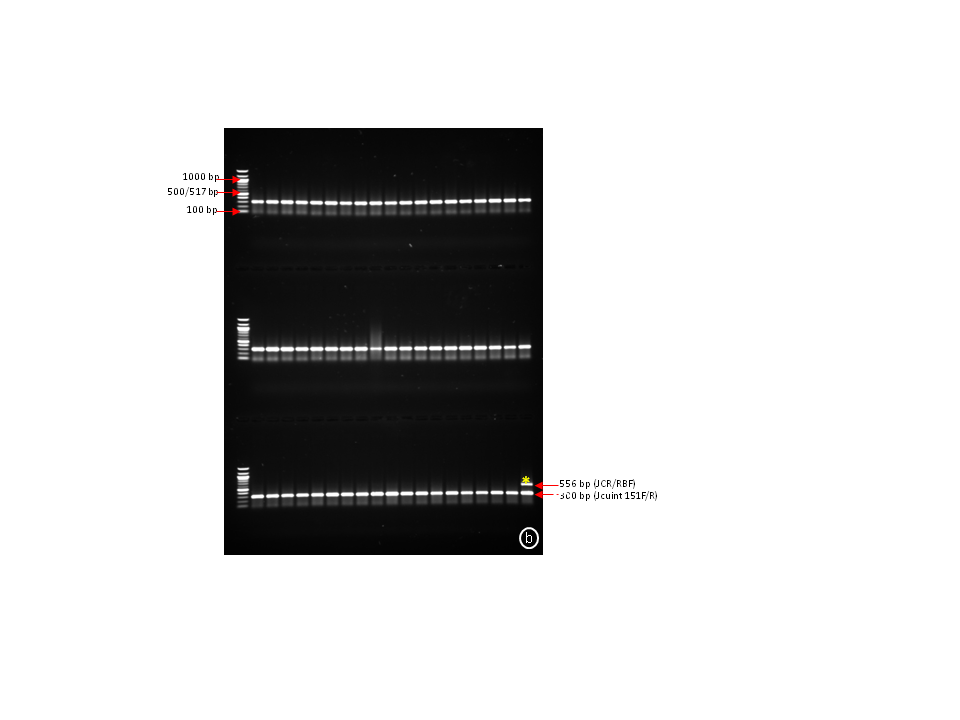

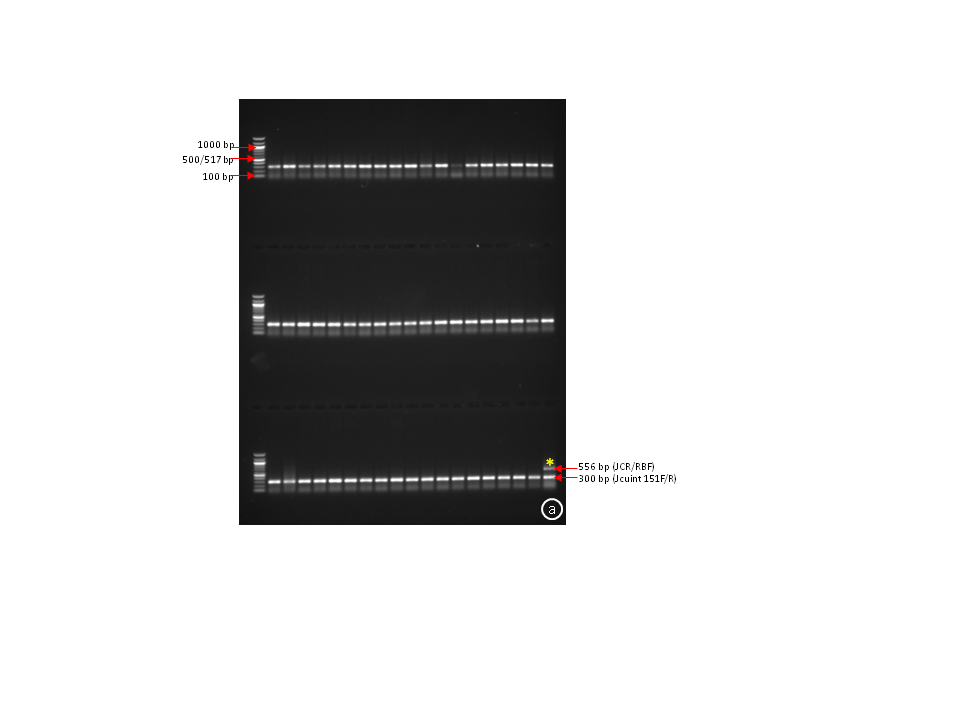


**Figure S4:** Multiplex transgene screening in seedlings from offsites non-transgenic plants by multiplex PCR with event specific primers (JCR/RBF) and an internal control primer pair ( 151F/R). a) PCR results for seedlings derived from non-transgenic plants located at 0.125 km, b) PCR results for seedlings derived from non-transgenic plants located at 2.0 km, c) PCR results for seedlings derived from offsite plants located at 2.5 km. * denotes positive control sample (non-transgenic spiked with X8#34 GM Jatropha leaf samples), 100 bp NEB ladder was used as size marker.


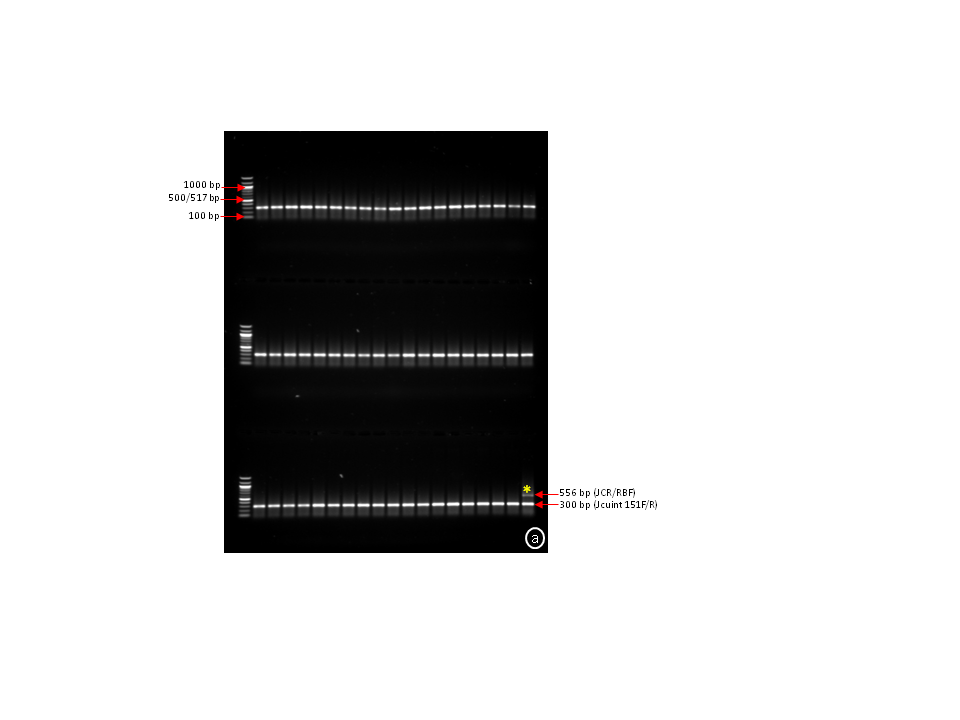

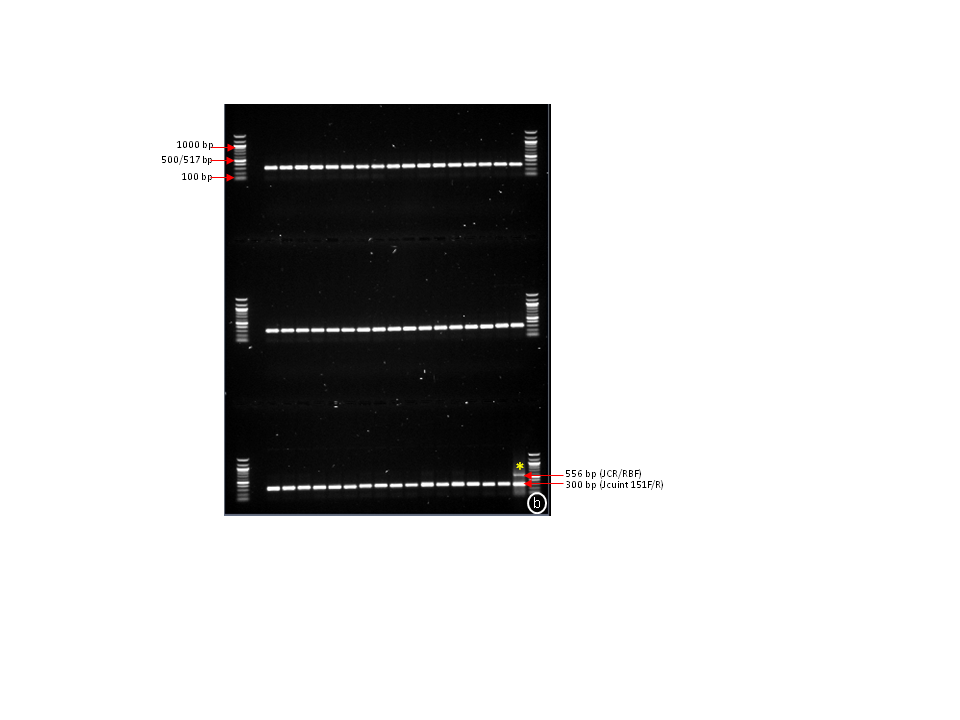


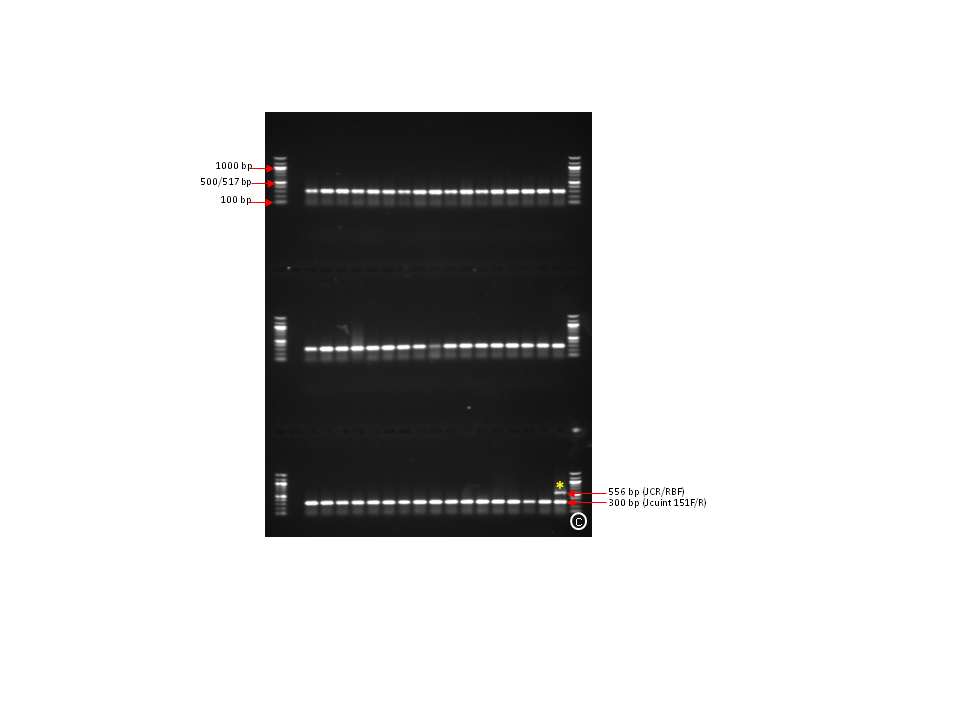


**Figure S5a:** Screening of transgene hybridization between X8#34 GM Jatropha with *J. integerrima*.

Lane M - 100 bp NEB marker, Lane 1-25 DNA samples from *J. integerrima*, Lane 26 Positive control *J. integerrima* leaf sample spiked with X8#34 GM Jatropha leaf sample.


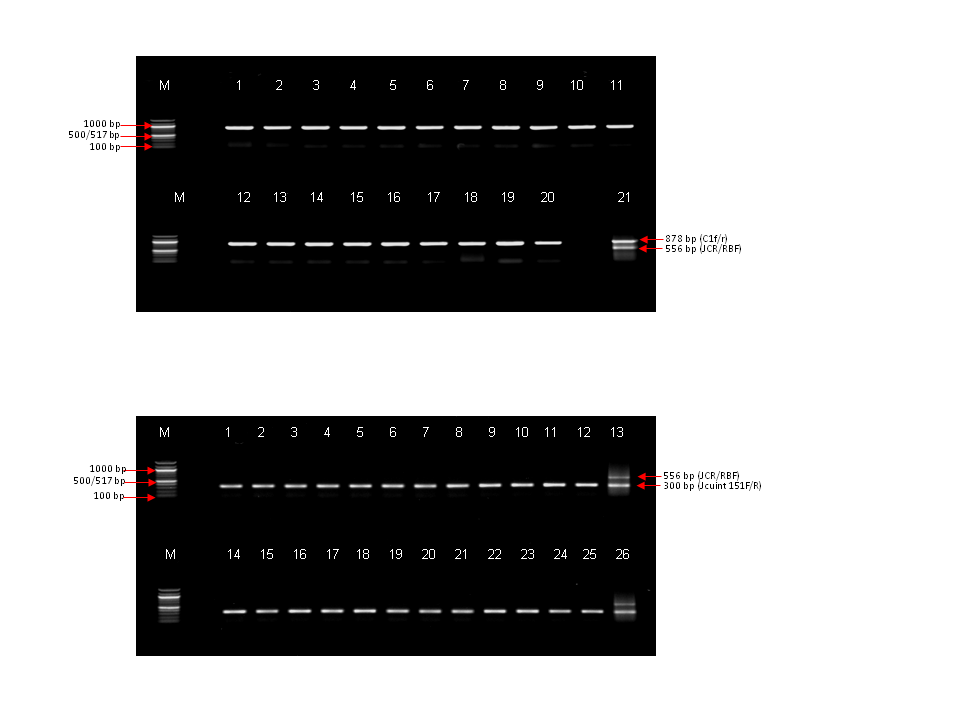


**Figure S5b:** Screening of transgene hybridization between X8#34 GM Jatropha with *J. integerrima*.

Lane M - 100 bp NEB marker, Lane 1-18 DNA samples from artificially pollinated *J. integerrima*, Lane 19 Positive control *J. integerrima* leaf sample spiked with X8#34 GM Jatropha leaf sample.


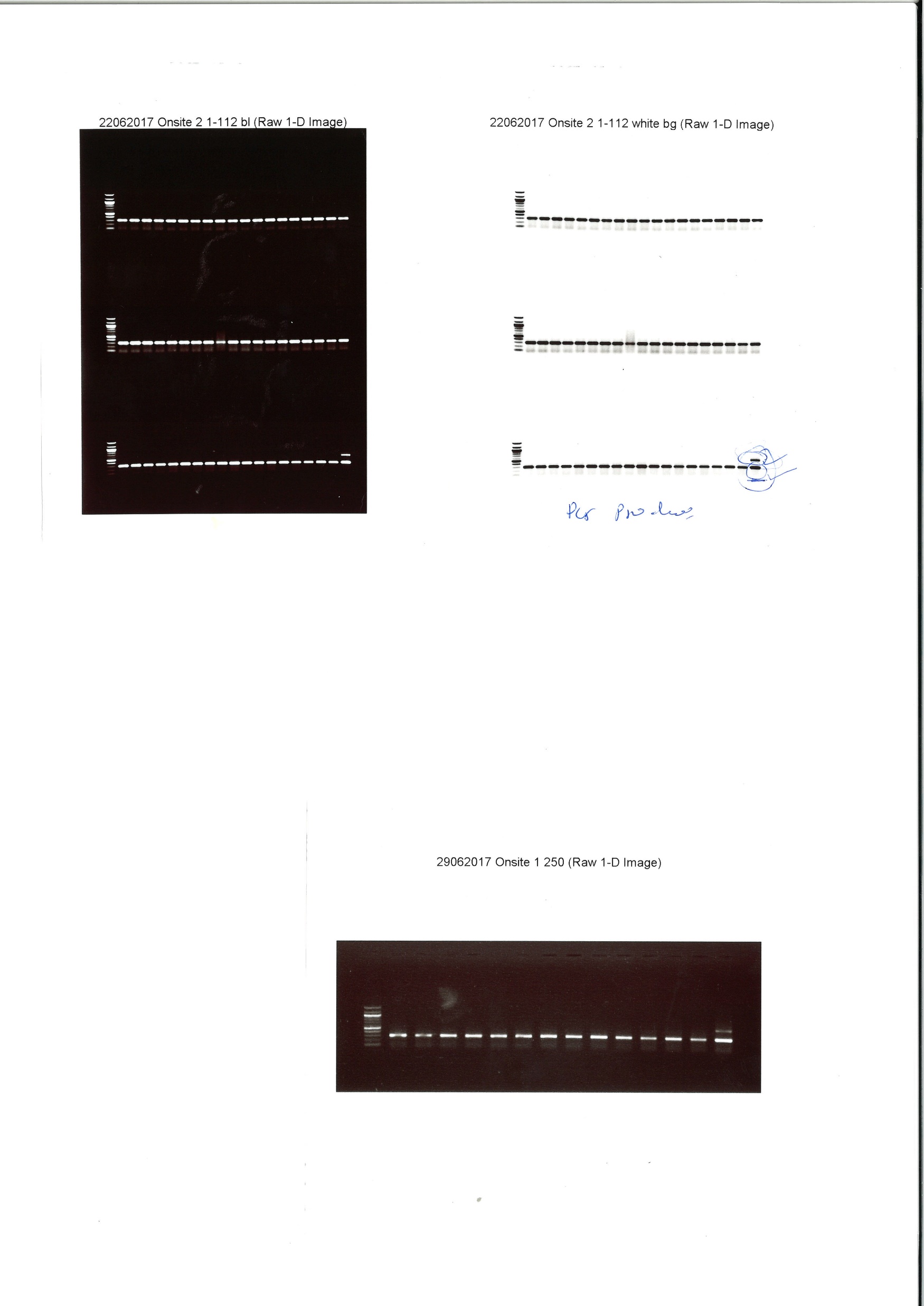


M 1 2 3 4 5 6 7 8 9 10 11 12 13 14 15 16 17 18 19

**Figure S6a*:*** Screening of transgene flow from X8#34GM Jatropha to weedy relative (*E.* *hirta*).

Lane M - 100 bp NEB marker, Lane 1-20 DNA samples from naturally pollinated *E. hirta*, Lane 21 Positive control *E. hirta* leaf sample spiked with X8#34GM Jatropha leaf sample.


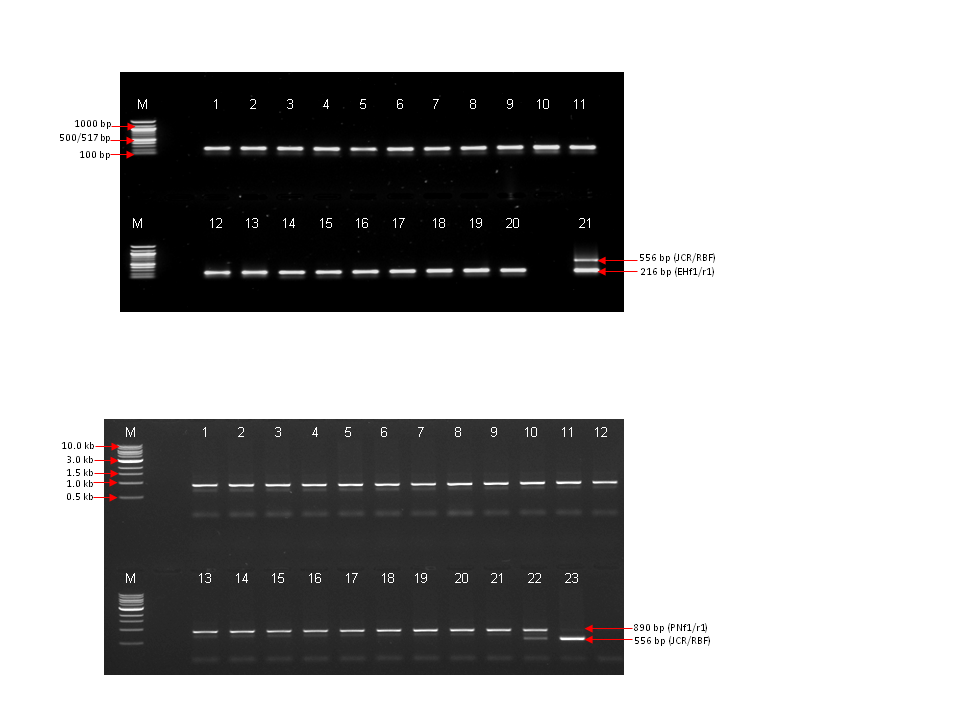


**Figure S6b*:*** Screening of transgene flow from X8#34 GM Jatropha to weedy relative (*E.* *hirta*).

Lane M - 100 bp NEB marker, Lane 1-11 DNA samples from naturally pollinated *E. hirta*,


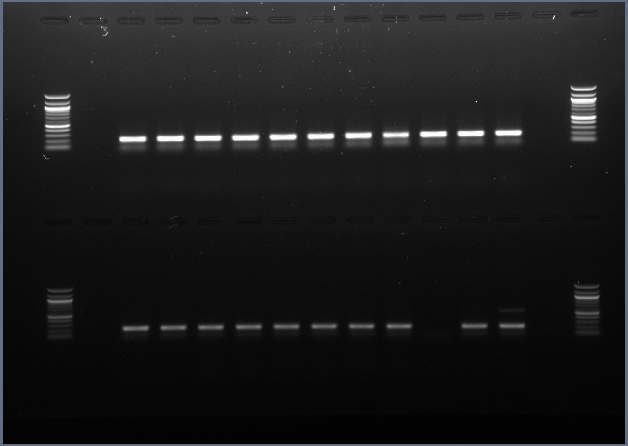


M 1 2 3 4 5 6 7 8 9 10 11

**Figure S7a:** Screening of transgene flow from X8#34 GM Jatropha to weedy relative (*P. niruri*).


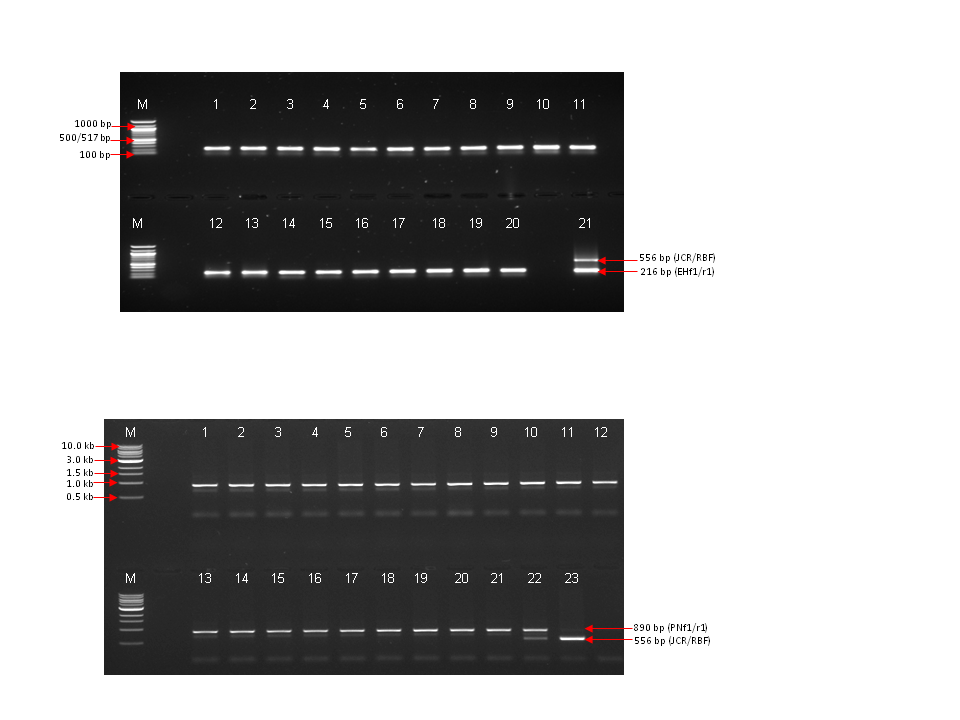
Lane M - 1 kb NEB marker, Lane 1 - 21 DNA samples from naturally pollinated *P. niruri*, Lane 22 Positive control *P. niruri* leaf sample spiked with X8#34 GM Jatropha leaf sample, Lane 23 DNA samples from X8#34GM Jatropha.

**Figure S7b:** Screening of transgene flow from X8#34 GM Jatropha to weedy relative (*P. niruri*).

Lane M - 1 kb NEB marker, Lane 1 - 20 DNA samples from artificially pollinated *P. niruri*, Lane 21 Positive control *P. niruri* leaf sample spiked with X8#34 GM Jatropha leaf sample.


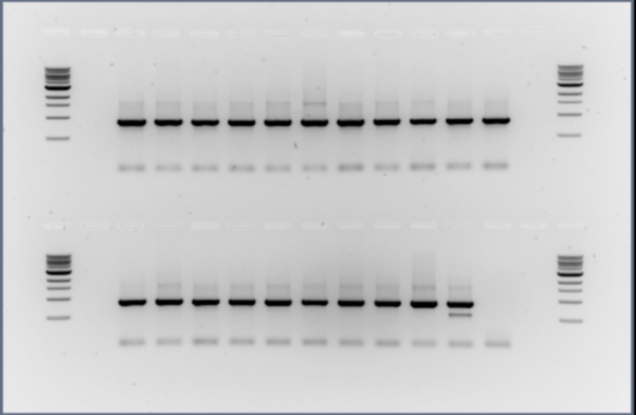


M 1 2 3 4 5 6 7 8 9 10 11

M 12 13 14 15 16 17 18 19 20 21

**Figure S8a*:*** Screening of transgene hybridization between X8#34GM Jatropha with a distant relative (Castor).

Lane M - 100 bp NEB marker, Lane 1-20 DNA samples from naturally pollinated Castor, Lane 21 Positive control Castor leaf sample spiked with X8#34GM Jatropha leaf sample.


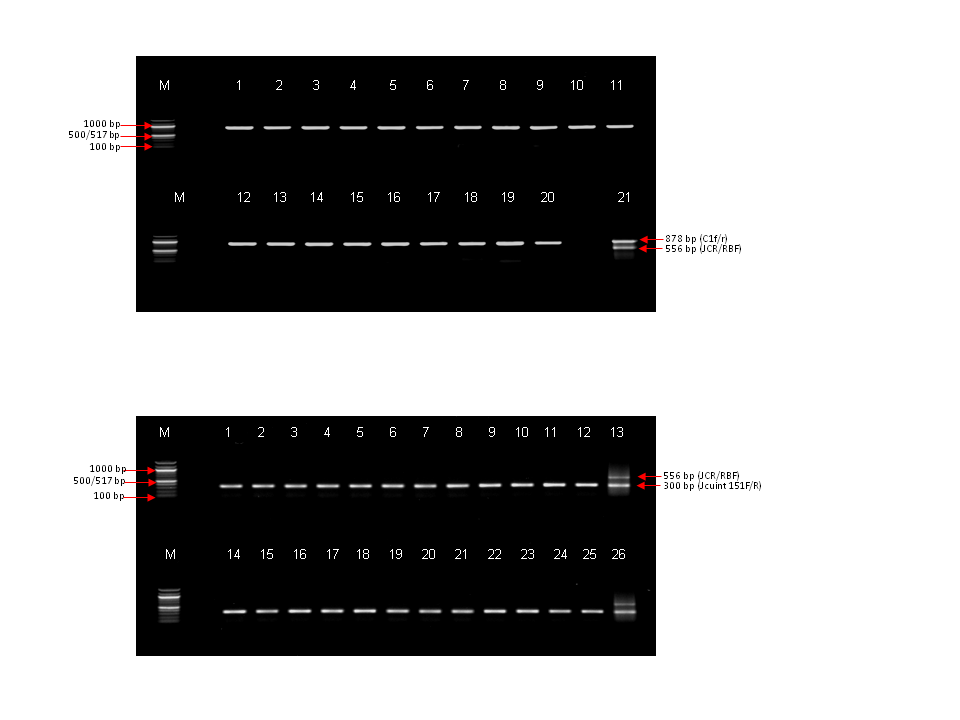


**Figure S8b*:*** Screening of transgene hybridization between X8#34 GM Jatropha with a distant relative (Castor).

Lane M - 100 bp NEB marker, Lane 1-20 DNA samples from artificially pollinated Castor, Lane 21 Positive control Castor leaf sample spiked with X8#34 GM Jatropha leaf sample.


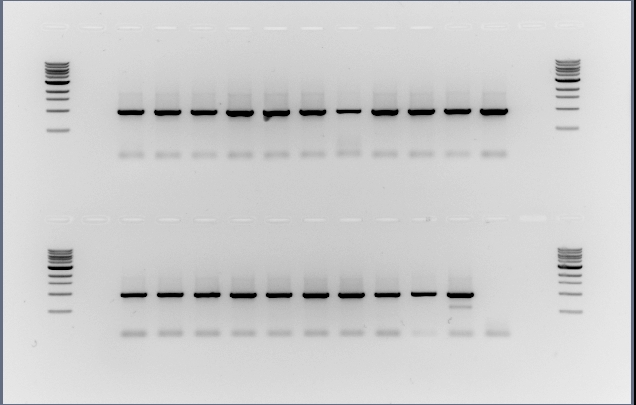


M 1 2 3 4 5 6 7 8 9 10 11

M 12 13 14 15 16 17 18 19 20 21
